# Host Genotype and Precipitation Influence of Fungal Endophyte Symbiosis and Mycotoxin Abundance in a Locoweed

**DOI:** 10.3390/ijms20215285

**Published:** 2019-10-24

**Authors:** Wei He, Linwei Guo, Lei Wang, Qianqian Zhao, Lizhu Guo, Wei Cao, Luis A. J. Mur, Yahui Wei

**Affiliations:** 1 Key Laboratory of Resource Biology and Biotechnology in Western China (Northwest University), Ministry of Education, College of Life Sciences, Northwest University, Xi’an 710069, China; hewei.scu@gmail.com (W.H.);; 2 School of Chemical Engineering, Northwest University, Xi’an 710069, China; 3 College of Urban and Environmental Science, Northwest University, Xi’an 710069, China; 4 Department of Grassland Science, China Agricultural University, Beijing 100083, China; 5 Institute of Biology, Environmental and Rural Science, Aberystwyth University, Aberystwyth SY23 3FL, UK

**Keywords:** *Oxytropis ochrocephala*, genetic variation, endophyte, *Alternaria oxytropis*, swainsonine

## Abstract

Many plant endophytes produce mycotoxins, but how host genetic variation influences endophyte colonization and mycotoxin production under natural conditions is poorly understood. This interaction has not been fully considered in many previous studies which used controlled experiments with agronomic or model plant species. Here, we investigated this interaction in a naturally occurring forb (a locoweed species) *Oxytropis ochrocephala*, its symbiotic endophyte *Alternaria oxytropis*, and the mycotoxin swainsonine. Host genetic variation was characterized by microsatellite markers. Endophyte infection rate and swainsonine levels were determined by PCR and HPLC, respectively. Genetic markers defined two distinct host populations and revealed that host genetics were significantly correlated with geographical location, elevation, and precipitation. As the host diverged, symbiotic interactions were reduced or failed to produce detectable swainsonine in one host population. Host genotype and precipitation had a significant impact in shaping swainsonine production at the population level. This study highlights the effect of host genotype in influencing this interaction in locoweeds.

## 1. Introduction

Fungal endophytes are ubiquitously associated with almost all plant species. These associations represent 400 Myr of co-evolution from when plant ancestors colonized and started to adapt to terrestrial ecosystems [1,2,3]. Although they have been considered as a synonym for mutualism, plant fungal endophytes can also be parasitic and/or commensal in different hosts and environmental contexts [4]. 

Plant fungal symbiosis is commonly considered mutualistic largely because in many cases such interactions improve fitness through increased growth and reproduction, abiotic stress tolerance, or herbivore deterrence [2]. Herbivore deterrence is often directly or indirectly associated with fungal-synthesized bioactive alkaloids such as ergot alkaloids, indole-diterpenes, lolines, and peramine [5]. 

The variety of toxic alkaloids and the extent of harmful effects on herbivores may be determined by both host and endophyte genotypes [6,7]. Indeed, the host genotype could be an instrumental factor in shaping the entire plant–fungal microbiome [8]. However, most studies examining the influence of the host genotype have used limited numbers of uncharacterized landraces, often of limited geographical origin. Such work, although important, cannot be easily translated to ecologically relevant situations where varied plant genotypes are exposed to a wide range of endophytic species under different environments. Moreover, most previous studies focused on agronomic [9] or model [8] plant species. For example, the interaction between plants and endophytic fungi has been extensively studied between cool-season gramineous grasses (e.g., *Lolium perenne*) and *Epichloё* (anamorph *Neotyphodium*) species, largely because such symbiosis makes the grass poisonous to herbivores and, thus, is detrimental to agriculture. However, this endophyte–grass interaction has also provided an ideal model for ecologists who are focusing on co-evolution and agronomists interested in forage improvement. Such studies have provided real insights into the fundamental underlying mechanism but their relevance to other common grassland species needs to be defined [10]. Additionally, many studies employed controlled experiments that seldom mimic realistic and variable field conditions [4]. Thus, assessments of the impact of host genetic variation on endophyte symbiosis requires that research should now involve a wider phytobiome community using non-model plant–fungi systems. These will provide a fuller understanding of plant–fungi co-evolution and, indeed, how these could be better exploited in agriculture practice. 

*Oxytropis ochrocephala* is a perennial native forb widely distributed in western China [11]. It forms a symbiotic interaction with the endophytic fungus, *Alternaria* sect. *oxytropis* (Ascomycota) [12,13,14], which produces the toxic alkaloid swainsonine making the plant poisonous to sheep and horses [15]. *A. oxytropis* is categorized as a Class 1 endophyte [1,16] as it infects a narrow host range of locoweeds (poisonous *Oxytropis* and *Astragalus* spp.) and is strictly vertically transmitted [17]. *A. oxytropis* has been posited as commensal [16] leads to no noticeable enhancement of the growth and reproduction of the forbs, or conferring of stress tolerance, or improving the host by acting as a deterrent to mammalian herbivores. Moreover, unlike in Poaceae where herbivore-toxic alkaloids can be stimulated by herbivore-induced tissue damage, *A. oxytropis* and swainsonine content does not increase following mechanical damage (e.g., clipping) of the plant, so, any defensive response in locoweeds is not likely to be improved by this symbiosis. 

Currently, the role of the host genotype in influencing the initial interaction is unclear. Swainsonine concentration in a host plant depends partially on the abundance of *A. oxytropis* [18]. Additionally, concentrations can reflect whether infection involves one of two classes of *A. oxytropis* strains (chemotypes), producing either high or low levels of swainsonine. Thus, the level of swainsonine production in subsequent generations can be influenced by the abundance and types of different chemotypes [19]. Cross-inoculation of two *A. oxytropis* chemotypes onto two endophyte-free locoweed accessions showed no noticeable effect on the amount of swainsonine produced. Although the authors suggested no role for host genotype on swainsonine production, the role of host genotypes in sorting the endophyte from different genetic backgrounds or chemotypes in the next generation has not been explored.

In this paper, we consider how *O. ochrocephala* has evolved, and whether host genotype influences endophyte colonization and mycotoxin production in naturally evolved populations. Additionally, we consider how environmental variables could be an important additional determining factor. Thus, we quantified plant genetic variation, endophyte infection rate, and mycotoxin concentrations in the wild Chinese forb *O. ochrocephala* from sites across western China. 

We demonstrate that while the host had diverged into two populations, host genotype influenced the host’s ability to support swainsonine-producing endophytes. In turn, swainsonine levels were affected by both host genetics and environment. For host genetic variation and swainsonine production, precipitation was suggested to be the major environmental variable. 

## 2. Results

### 2.1. Genetic Variation in O. ochrocephala

Fourteen polymorphic EST-SSR (Expressed Sequence Tags- Simple Sequence Repeats) primer sets were used to characterize genetic diversity in 368 *O. ochrocephala* samples from 33 accessions gathered from western China. Genetic diversity was calculated at 14 loci (Table S1A) and 192 alleles were identified and the mean allele number per locus was 13.7 (5~25). The mean effective allele number was 5.23 (1.94~8.73) due to the large number of rare alleles detected (100 out of 198). The average observed heterozygosity (*Ho*) was 0.474 (0.3016~0.652) whereas the expected heterozygosity (*He*) was 0.766 (0.484~0.887). The average polymorphic information content (*PIC*) was 0.73 (0.41~0.82), which indicated a satisfactory discriminative power for the primer sets. *F* statistics inbreeding coefficients were F_IS_: 0.176, F_ST_: 0.253, and F_IT_: 0.385. The overall gene flow (*Nm*) equaled 0.738 < 1. This low gene flow indicated limited gene flow amongst the sampled accessions. 

**Figure 1:**
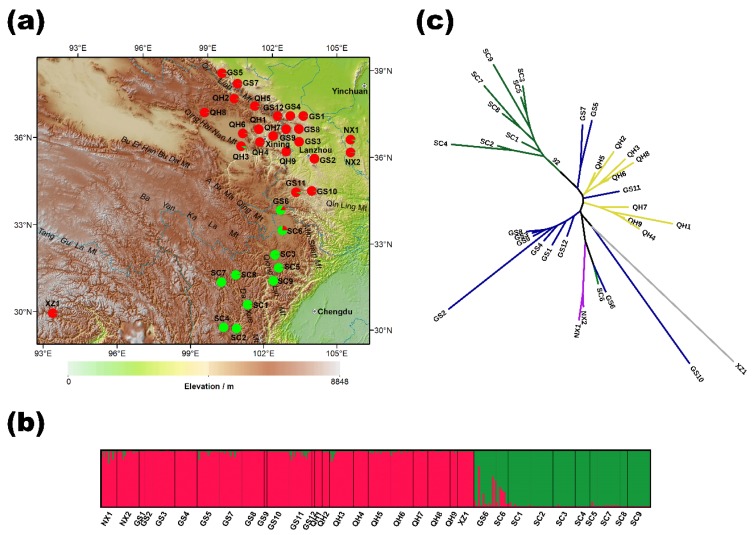
Genetic variation and differentiation in the 33 *Oxytropis ochrocephala* accessions in western China revealed by 14 SSR (Simple Sequence Repeats) loci. Accession code can be referred to in Table S2A. (**a**) Histogram of the STRUCTURE genetic assignment of the 33 accessions. (**b**) Geographic origin of the 33 accessions and their color-coded genetic partitioning at the most likely (K = 2). Red and green correspond to the genetic assignment by STRUCTURE (in panel **b**). The horizontal length of each color represents the probability of the individual within that cluster. (**c**) Neighbor-joining dendrogram of the 33 accessions. Different colors of the branch indicate samples collected from the five provinces.

STUCTURE analysis was used to reveal the genetic structure of the sampled *O. ochrocephala*. The model value of ΔK was found to be at K = 2, suggesting that genetic differentiation is best explained if two distinct, large-scale populations were assumed to exist (Figure S1). Under K = 2, all samples were assigned by their partition into the two populations (represented by green and red in Figure 1a). Accessions were linked with their geographical locations and the percentage of genetic assignment between the two populations is shown (Figure 1c). Accessions from Ningxia (NX), Gansu (GS, except GS6), Qinghai (QH), and Tibet (XZ) shared similar genetic homogeneity (shown in red) and were classified as population 1 (pop 1). Based on the same rationale, accessions in Sichuan, including SC6, and accession GS6 (two of the accessions of SC6 and GS6 that were geographically isolated from population 1 by the Qinling Mountain) formed population 2 (pop 2, shown in green). The genetic structure of *O. ochrocephala* was further demonstrated by genetic-distance-based phylogenetic analysis. A neighbor-joining (NJ) dendrogram constructed based on Nei’s genetic distance showed a consistent pattern of genetic relationships (Figure 1b). In the NJ dendrogram, all the accessions in Sichuan (except SC6) formed a single cluster from the four provinces from the north, with a strong bootstrap value (92). However, the probability-based STRUCTURE method and genetic-distance-based NJ method suggested some genetic relatedness of the accessions of GS6 and SC6. In this latter analysis, GS6 and SC6 are genetically closer to cluster 1. These may encompass the genetic complexity of the two populations or could represent a third population that was not sufficiently resolved by our 14 SSRs (Simple Sequence Repeats). Although GS6 and SC6 are located between the two well-separated populations and appeared to be a genetic mixture of the two, they could also represent an intermediate genetic lineage during evolution (e.g., evolution happened from the north to the south via regions comprising GS6 and SC6). Thus, we performed an evolutionary route analysis using the DIYABC (Do It Yourself Approximate Bayesian Computation) software (Figure S2). Results suggested that, of the four proposed scenarios, the most likely (posterior probability = 0.9557) was that pop 1 was the common ancestor of all the investigated accessions. This proposed that pop 1 evolved and then diverged further into clades containing accessions GS6 and SC6 and the other, the pop 2 accessions. Pop 1 and 2 do not share much genetic collinearity. 

Genetic variation in *O. ochrocephala* was further quantified at different hierarchical levels. Where STRUCTURE analysis indicated two genetically distinct clusters (i.e., K = 2), analysis of molecular variance (AMOVA) showed that 72.36% of the total genetic variation could be attributed to “within accession” variation, 17.95% to “amongst accessions within populations” and 9.69% to “amongst populations” variation (Table 1). The fixation indices showed that genetic differentiation at all the three hierarchical levels were highly significant (*Fst* = 0.276, *Fct* = 0.0969, and *Fsc* = 0.199, *p* < 0.0001). 

### 2.2. Endophyte Infection Rate and Swainsonine Concentrations

**Figure 2:**
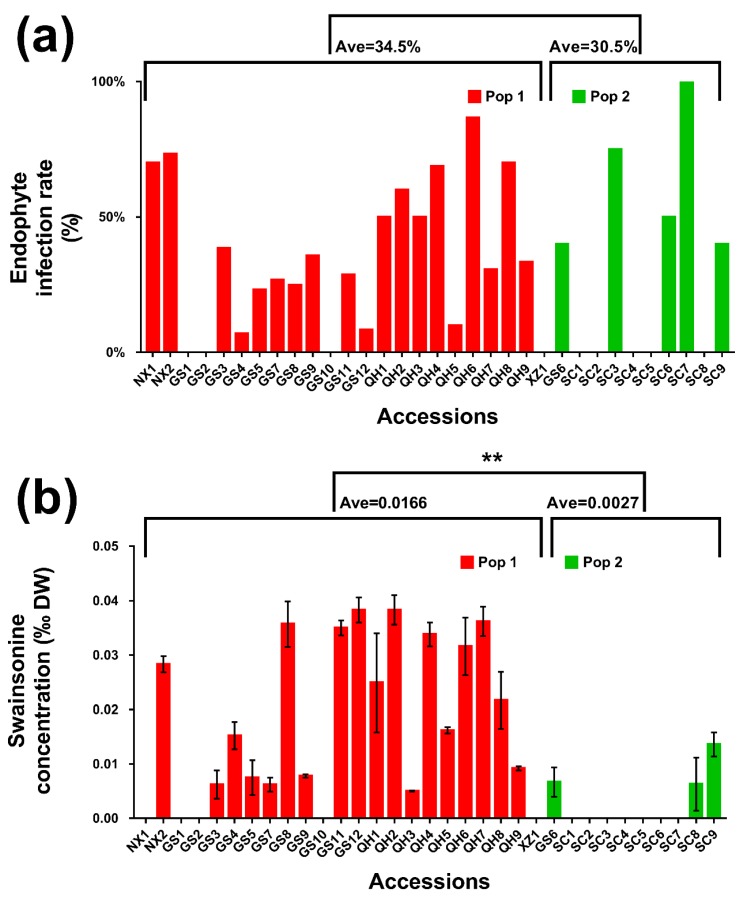
Endophyte (**a**) and swainsonine (**b**) concentration in 33 accessions of *O. ochrocephala.* Pop 1 (population 1) represents accessions from Ningxia (NX), Gansu (GS, except GS6), Qinghai (QH), and Tibet (XZ) and pop 2 represents accessions from Sichuan (SC) and GS6. Bars represent mean ± SEM in (**b**). Asterisks indicate significant difference.

In order to compare the ability to support endophyte symbiosis and to produce swainsonine in host plants, we measured endophyte infection rates and swainsonine concentration in each accession (Figure 2 and Table S3). There is no significant difference in endophyte infection rate in population 1 (34.5%, *n* = 20) and 2 (30.5%, *n* = 10), but swainsonine concentrations differed significantly (0.0166‰, *n* = 20 in pop 1 and 0.0026‰, *n* = 10 in pop 2). 

### 2.3. Interaction between Genetics and Environment and Their Integrated Effect on Endophyte Symbiosis and Swainsonine Production

We tested whether environment (represented by geographical locations and elevation, and deduced annual precipitation and temperature) plays an important role in plant genetic variation. Additionally, we tested whether G x E (genetic x environment interactions) acted to influence patterns of endophytic symbiosis and mycotoxin biosynthesis. Therefore, we performed a series of pairwise correlation analyses between each two factors from the environmental and genetics, and endophyte infection rate and swainsonine concentration. Results showed that geographical distance (*r* = 0.523, *p* < 0.001, and *n* = 528 pairwise, Table 2 and Figure S3a) and elevation (*r* = 0.424, *p* < 0.001, and *n* = 528 pairwise, Table 2, Figure S3b) strongly correlated with genetic distance. At a higher genetic level, elevation also significantly correlated with population partition (*r* = 0.606, *p* < 0.001, and *n* = 33) and population clustering (*r* = 0.665, *p* < 0.001, and *n* = 33). This demonstrated the effect of isolation-by-distance and altitudinal environmental variation on the genetic divergence seen in the two populations of *O. ochrocephala*.

**Figure 3:**
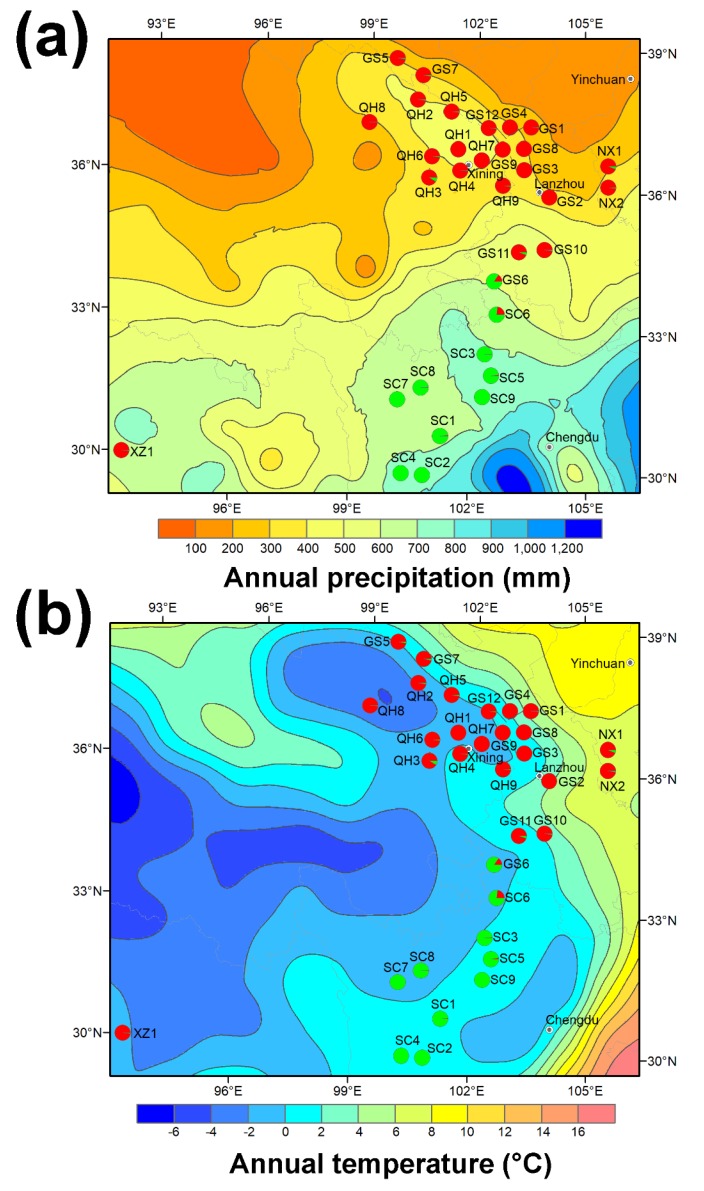
Annual precipitation (**a**) and temperature (**b**) in the 33 *O. ochrocephala* accessions and their genetic partition by the STRUCTURE analysis.

Altitudinal environmental variation is often linked with changes in temperature and precipitation. Across our sampling sites, both annual temperature (*r* = −0.489, *p* < 0.01, and *n* = 33) and precipitation (*r* = 0.730, *p* < 0.001, and *n* = 33) changed with elevation (Table 2, Figure 3). Pop 1 had drastically lower (*p* < 0.001) average annual precipitation (392 mm) than pop 2 (694 mm). The correlation coefficients between annual precipitation and genetic distance (*r* = 0.4836, *p* < 0.001, and *n* = 528 pairwise), population partition (*r* = 0.584, *p* < 0.001, and *n* = 33), and clustering (*r* = 0.762, *p* < 0.001, and *n* = 2) were significant at each level of genetic hierarchy. Thus, we suggest that precipitation plays a key role in driving plant genetic divergence in *O. ochrocephala*. Such patterns were not observed in annual temperature, though it was moderately correlated with expected accession heterozygosity (*r* = −0.371, *p* < 0.05). Higher temperature may therefore influence the reproduction of *O. ochrocephala* by changes in flowering, pollination, and the setting of seeds, which can contribute to heterozygosity levels.

In terms of endophyte colonization, the data showed that at the accession level, host genetics and environment are not correlated with the endophyte infection rate of *A. oxytropis* (Table 3). Nevertheless, comparison of endophyte infection rate and swainsonine concentration in the two populations (Figure 2) showed that, in pop 2, 50% of the accessions had no *A. oxytropis* infection (compared to 5% in pop 1), and also 30% of the accessions with *A. oxytropis* symbiosis had no detectable swainsonine (10% in pop 1). Chi-square test of the distributions of the four types of endophyte/swainsonine combination (Endo+ Toxic+/Endo+ Tox−/Endo− Tox+/Endo− Tox−) are not equal in the two populations (*p* = 0.0095). This suggests that in the two populations, the ability to produce swainsonine by endophytes may shift toward being more stochastic in pop 2.

Although the correlation between swainsonine and host genetics is not strong when considering genetic distance (*r* = 0.218, *p* < 0.001, and *n* = 450 pairwise), it became more obvious at higher levels of genetic divergence (r = 0.492, *p* < 0.01 with population partitioning and *r* = 0.570, *p* < 0.01 with clustering, *n* = 30, Table 3). With regard to environmental factors, swainsonine was negatively correlated with annual precipitation (*r* = −0.444, *p* < 0.05, *n* = 30) and also correlated with geographical distance (*r* = 0.340, *p* < 0.001, and *n* = 450 pairwise). A further multiple regression analysis showed that the three significantly correlated variables (geographical distance, precipitation, and genetic partition) explained 39% (*r* = 0.626, *p* = 0.006) of the total variation in swainsonine concentration. Taken together, these data indicate that G x E affects the production of swainsonine.

## 3. Discussion

In this study, we examined the genetic variation in a wild locoweed plant *O. ochrocephala* and determined its impact on endophyte symbiosis and mycotoxin biosynthesis under variable environmental conditions. In assessing such relationships, *O. ochrocephala* represents a useful model as it has not been even semi-domesticated, as is the case with the forage grass *L. perenne,* and, therefore, can better reflect natural conditions. To elucidate the influence of genetic variation in *O. ochrocephala* on endophyte symbiosis and mycotoxin biosynthesis, we employed SSR markers. Overall genetic variation indicated by the expected heterozygosity across 14 loci was high (*He* = 0.762). Around three-quarters of this genetic variation could be attributed to within-accession variation and the rest among accessions or populations (F_ST_ = 0.276). This accorded with the pattern of genetic variation seen in *O. campestris* var. *chartacea* (Fassett’s locoweed) in the United States [20]. The overall heterozygosity observed was much lower than expected, suggesting inbreeding occurred within and amongst the accessions, as also indicated by the *F* statistics. As the overall genetic variation is high, inbreeding is likely to have occurred due to the geographical isolation of the surveyed accessions and accompanying limited gene flow (*N*m < 1). Low gene flow is consistent with the isolation-by-distance pattern based on the correlation between genetic distance and geographical distance. Interestingly, genetic distance is also correlated with elevation.

Previous studies indicated that in plants the median F_ST_ for mixed breeding or selfing/clonal propagation is 0.18 and 0.36 (corresponds to median *N*m of 1.17 and 0.45), respectively [21]. The F_ST_ (0.253) and *N*m (0.738) in *O. ochrocephala* fall in between the above two, indicating both mixed breeding and clonal strategies are employed. Although *Oxytropis* spp. are generally considered to be cross pollinated, some species can be self-compatible [22].

STRUCTURE analysis showed that the surveyed accessions of *O. ochrocephala* split into two genetically distinct populations. AMOVA analysis suggests significant genetic differentiation at three hierarchical levels: within accession, among accession, and between the two genetic populations. The two populations were allopatrically isolated by the Qinling Mountains (Figure 1a), and most accessions are distributed along Qi Lian, Qing Hai Nan, Min, Qionglai, and Da Xue mountains. These mountains represent barriers that effectively prevent any gene flow so that they could result in the genetic divergence. As *Oxytropis* is considered to have a long evolution history [23], we suggest that the high genetic diversity we observed with *O. ochrocephala* reflects the fragmentation of the founder population.

To date, the ecological role of *A. oxytropis* in locoweeds is poorly understood. Since *A. oxytropis* negative individual plants were found in almost every accession, we suggested that there was no absolute requirement for this endophyte’s presence in *O. ochrocephala*. This agrees with previous suggestion that this endophyte does not improve the fitness of the host as a commensal endophyte [16]. Nevertheless, swainsonine-producing endophytes may modulate endophyte community richness. A recent study found that the abundance of the endophyte *Alternaria fulva,* a swainsonine-producing endophyte in a sibling locoweed species *Astragalus lentiginosus*, negatively correlates with α- but not β-diversity of the foliar endophyte community [24].

In pop 2, several accessions had completely lost *A. oxytropis*. A previous study found that although endophyte transmission rates to seeds can be 100%, in 15% these endophytes were of low abundance [17]. This pattern is likely to occur in successive generations resulting in a cumulative reduction/loss of endophytes in those accessions in pop 2. Previous studies in grasses suggested that in a strictly vertically transmitted and asexual endophyte, host features (which encompass defined cellular structures, biochemistry, patterns of defense, or key receptors) are needed to support endophytic colonization [25,26]. Genotypes that do not possess these features or lose them through segregation or genetic recombination will lead to a loss of endophyte infection. This commonly occurs in established and newly diverged populations [27]. The DIYABC evolution route analysis showed that pop 2 was evolved from pop 1 and during this process, key loci to support endophyte symbiosis may have been lost. Moreover, in pop 2, accessions with high endophyte infection rate had no detectable swainsonine. Although, this appears to be counter-intuitive, this agrees with the high- and low-swainsonine producing chemotypes of endophytes described by Cook et al. [18]. In addition, we observed that one rare case (SC8) out of the 33 accessions produced swainsonine but no detectable endophyte, which suggests that other endophytes that could produce swainsonine may exist in this accession. Such a swainsonine-producing endophyte has been identified in a legume in China, e.g., *Alternaria gansuense* [28]. Other fungi that also produce swainsonine, such as *Metarhizium* sp., *Slafractonia leguminicola*, and ringworm fungi [29], have not yet been isolated from China. This rare case agrees with the recent finding that dominant but not prevalent *A. fulva* assemblies determine swainsonine concentration [24], therefore, in our case other strains may not have been amplified by the PCR primers.

These points notwithstanding, we found that swainsonine concentration correlated with host genetic variation, which suggests that host genotype affects the production of this mycotoxin. This apparently does not agree with a recent study which reports the cross inoculation of two endophyte strains with two plant accessions showing no noticeable effect of plant origin in *Oxytropis sericea* [18]. However, this study did not access genetic variation in the host and highlights the value of our wider-ranging study. We found great variation in swainsonine content even within the same host population, so that in earlier smaller studies the effect of host genotypes on swainsonine biosynthesis may have been missed.

We were also able to correlate geographical distance and annual precipitation with swainsonine concentration. In particular, precipitation is correlated negatively with swainsonine levels. A previous study in *O. sericea* showed that swainsonine is higher in plants subjected to drought stress [30]. Thus, plants under water stress in the northern population could result in increased production of swainsonine. Alternatively, the higher precipitation in the southern population may exert a long-term “tuning down” effect on swainsonine. Since geographic distance and precipitation are correlated with plant genetic variation, environment factors influencing swainsonine production may also act indirectly through plant genetic divergence. Future controlled experiments are needed to test this hypothesis.

The key findings from this study are that plant genetic variation has a key filter effect on holistic endophyte colonization and G x E on mycotoxin production. Since endophytic colonization and mycotoxin production in *O. ochrocephala* has neutral effects on host fitness, host genotype appears to be a major defining feature, most likely due to genetic drift. In our case, the G x E effect on swainsonine could reflect a combination of genetic and epigenetic effects [2].

In a recent study, the deterministic role of host genetics was not found in *A. lentiginosus*, where the abundance of the fungus *A. fulva*, rather than host genetic distance, predicted the leaf endophytic fungal diversity [24]. For our model system, we hypothesize that host genetics act on sorting swainsonine producing endophytes with wider impacts on the entire endophytic fungal community. To better understand these processes, further work is needed where the genomic-level variation, employing deeper sequencing depth, is extensively assessed in both host and endophytes in larger populations. Such information can be integrated with environmental data and mycotoxin levels for effective identification of genetic loci governing the interaction of hosts and endophytes.

## 4. Materials and Methods

### 4.1. Sample Collection and DNA Extraction

Leaf material of *O. ochrocephala* samples were collected during the flowering season of July to August from 33 localities across five provinces in China in 2012, 2013, and 2014. In total, 368 individuals were sampled (*n* = 2 to 16 per accession subject to availability, Table S1). Sample distribution spanned approximately five degrees by longitude and ten degrees by latitude, equivalent to 261,516.7 km² (Figure 1). All of the samples were collected and stored in airtight bags with silica gel until DNA extraction within two weeks. Voucher specimens from each accession were deposited in the Poisonous Weed Research Centre at Northwest University in Xian, China. Total genomic DNA was extracted from 50 mg dry leaves using a plant genomic DNA extraction kit (Tiangen, Beijing, China). DNA integrity was determined on 1% agarose gel and the concentration was measured on a NanoDrop ND-1000 Spectrophotometer (NanoDrop Technologies, Wilmington, DE, USA). DNA samples were aliquoted and stored at −20 °C until use.

### 4.2. Microsatellite Genotyping

EST-SSR (microsatellite) primers were designed based on transcriptome sequencing data previously generated in our laboratory [31]. Fourteen polymorphic and reproducible primer sets were selected and used for further genotyping after primer set screening (Table S2). Seven primers (forward only) were FAM labeled and the other seven were TAMRA labeled. PCR amplification was performed in a 20 μL volume using Premix Taq DNA Polymerase PCR Mix (Takara, Japan) with 10 ng DNA and primers at 0.3 μM. PCR reactions were performed as follows: 94 °C denaturation for 5 min; 94 °C denaturation 30 s, 54 °C annealing 35 s, and 72 °C extension 40 s, 35 cycles; and final extension at 72 °C 3 min. PCR products were diluted 10-fold and 1 μL was mixed with 15 μL loading buffer, and analyzed on an ABI 3730XL DNA sequencer (Thermo Fisher Scientific, Waltham, MA, USA). Electropherograms were analyzed and DNA fragment sizes deduced from molecular weight standards using GeneScan 4.0 and Gene Mapper 4.0 (Thermo Fisher Scientific), respectively. All scores were checked manually for accuracy.

### 4.3. Measuring Swainsonine Content

Swainsonine concentration was measured for each accession. Leaf samples of the same weight from every individual within one accession were pooled together and the levels of swainsonine measured. Swainsonine extraction was performed as previously described [32]. In brief, one gram of dried plant leaf material was extracted in five successive extractions of 10 mL of absolute ethanol with sonication for 2 h each. Supernatants were merged and dried in a rotavapor, and 1 M HCl was added to the dried extracts until they were dissolved. The solvent was extracted using n-butyl alcohol, then the non-organic phase was titrated using 10 % (*w*/*v*) NaOH to adjust the pH to 9–10. After centrifugation, n-butyl alcohol was added, and the solution was extracted eight times. The n-butyl alcohol phase residue was recovered, combined, and evaporated. The residue was dissolved in 1 mL methanol and filtered for further analysis. Three independent extractions were performed for each accession.

Swainsonine concentration was analyzed on a HPLC using a Hypersil ODS C18 (250 mm × 4.6 mm, 5 μm) column (Elite, Shanghai, China) according to the method of Duan et al. [33]. Pure swainsonine (Santa Cruz Biotechnology, Dallas, TX, USA) was dissolved in methanol and diluted to establish a standard curve. Twenty microliters of the standards and samples were injected. Elution solvent was 20 mmol/L KH_2_PO_4_ (pH 7.0) buffer/acetonitrile (*v*/*v*, 99:1). Flow conditions were 0.8 mL/min with the column maintained at room temperature. Swainsonine concentrations were calculated from peak areas obtained at 205 nm with reference to the standard peak area. The detection limit of swainsonine was 0.005 mg/mL. Swainsonine concentrations were calculated and represented as a millesimal of the dry weight of plant leaves (SW‰).

### 4.4. Determination of Endophyte Infection Rate

Although previously a quantitative real-time PCR method was established to determine the amount of endophyte in leaf material [34], we adapted it into a semi-quantitative method to determine *A. oxytropis* infection rate (see below for explanation). PCR was performed using ITS5 (5′-GGA AGT AAA AGT CGT AAC AAG G-3′) and OR1 (5′-GTC AAA AGT TGA AAA TGT GGC TTG G-3′) primers and PCR products were digested with AVA II endonuclease and separated on agarose gels. Crucially, the *A. oxytropis* ITS (Internal Transcribed Spacer) sequence can be digested by AVA II into ~380 and 200 bp size bands. As many amplicons could only be partially digested by AVA II, we randomly selected and sequenced 90 samples (approximately 25% of the total samples) to validate the identity of the PCR amplicons (Table S4). Amplicons that could be completely digested (e.g., Figure S4, sample A) were found to be *A. oxytropis* ITS sequence (573 bp, homologous to AY228650.1 isolated from locoweeds, Figure S6), whilst the undigested amplicons (e.g., Figure S4, sample B) represented a chloroplast sequence (560 bp, homologous to *Astragalus mongholicus* chloroplast gene, KU666554.1, Figure S7). In these 90 samples, when samples could be partially digested (e.g., Figure S4, sample C), they also had mixed peaks in sequencing, indicating they contained two amplicons of similar sizes. Thus, for those samples we sequenced the two digested amplicons (~380 and 200 bp, respectively). Sequence alignment of the digested bands suggested those partially digested amplicons represented both the *A. oxytropis* ITS sequence (Figure S5) and the chloroplast sequence and was, therefore, considered an *A. oxytropis*-positive plant. In all the 90 samples sequenced, no other *Alternaria* species were identified despite their high genetic homology to *A. oxytropis* [35]. Infection rates reflected the number of infected individuals as a proportion of the total number of plants in each accession.

### 4.5. Data Analysis

Genetic diversity was calculated for all loci using POPGENE version 1.31 [36] including the observed number of alleles (*Na*), the effective number of alleles (*Ne*), and the expected (*He*) and observed (*Ho*) heterozygosities, inbreeding coefficient (*F* statistics), and polymorphic information content (*PIC*).

AMOVA analyses and differentiation index (*Fst*) were performed using Arlequin version 3.11 [37]. A phylogenetic tree based on Nei’s 1972 genetic distance method [38] was constructed using the neighbor-joining (NJ) method in Software PowerMarker version 3.25 [39]. Bootstrap values were calculated using 1000 replicates. To infer the genetic structure of the accessions, individual-based population assignment was calculated to identify differentiated genetic clusters using the Bayesian assignment clustering method implemented in STRUCTURE version 2.3.4 [40]. Potential genotype clusters (K) from 1 to 15 were assumed under the admixed model and the assumption of correlated allele frequencies among populations. For each value of K, 50 runs were performed with 100,000 iterations discarded as burn-in followed by an additional 1 million MCMC (Markov Chain Monte Carlo) iterations. The best number of clusters (no. of K) was identified when LnP (D) values reached a plateau, indicating a negligible increase in LnP (D) in the next K [41]. STRUCTURE analysis results were displayed using *DISTRUCT* [42]. Evolutionary route analysis was performed using DIYABC [43].

Pairwise genetic distances between all accessions were calculated using GenAlEx version 6.5 [44]. Correlations between genetic distances [45] and geographic distance/elevation distance were determined using Isolation by Distance web service [46]. In order to determine the influence of environmental factors, we obtained raw daily precipitation and temperature data (1970–2010) from the China Meteorological Administration and calculated annual precipitation and temperature using the Kriging interpolation method in ArcGIS (ESRI (Environmental Systems Research Institute), Redlands, CA, USA). Mantel tests were performed to determine the correlation of genetic distance and geographical distance against other variables using TFPGA (Tools For Population Genetic Analyses) version 1.3 [47] after converting the data into matrices [48]. Other pairwise correlation tests were performed in SPSS (IBM (International Business Machines), Armonk, NY, USA) and data were represented by Pearson’s correlation coefficient, or Spearman’s rho when involved with non-normally distributed genetic partition and cluster data. To reduce bias from small sample size (*n* < 5), accession GS1, GS10, and QH1 were excluded from the correlation analyses relating to endophyte infection rate and swainsonine concentration. Multiple regression tests were also performed in SPSS, in which swainsonine levels were assigned as response variable and all the others with significant association assigned as explanatory variables. Differences in the means of the two populations were analyzed in SPSS by one-way ANOVA.

We further wanted to infer how the ability to produce swainsonine varied during host divergence, therefore, we compared in each accession the endophyte infection rate and swainsonine content. Based on whether they contain endophyte and swainsonine or not, all the accessions were classified as four types: Endophyte+ Toxic+/Endo+ Tox−/Endo− Tox+/Endo− Tox−. To determine whether the distributions of the frequencies of the four types in the two populations are equal a Chi-square test was performed in SPSS.

## Figures and Tables

**Table 1:** Analysis of molecular variance (AMOVA) for *O. ochrocephala* at fourteen SSR loci at the following hierarchy: (1) amongst the two populations identified by STRUCTURE analysis; (2) amongst the 33 accessions within the two populations; and (3) within accessions.

Source of Variation	*d.f.*	Sum of Squares	Variance Components	Percentage of Variation	Fixation Indices
Amongst populations	1	173.928	0.505 Va	9.69	*Fct* = 0.0969 ***
Amongst accessions within populations	31	757.888	0.937 Vb	17.95	*Fsc* = 0.199 ***
Within accessions	703	2653.730	3.775 Vc	72.36	*Fst* = 0.276 ***
Total	735	3585.546	5.217	100	

Asterisks (***) indicates significant value (*p* < 0.001).

**Table 2:** Environmental effects on plant genetic divergence.

Environment	Elevation	Genetics			
		Genetic Distance	Population Partition	Cluster	He
Geographic distance	---	*r* = 0.523 ***	---	---	---
Elevation	---	*r* = 0.424 ***	*r* = 0.621 ***	*r* = 0.662 ***	*r* = 0.010
Annual precipitation	*r* = 0.730 ***	*r* = 0.484 ***	*r* = 0.584 ***	*r* = 0.762 ***	*r* = −0.256
Annual temperature	*r* = −0.489 **	*r* = 0.258 *	*r* = −0.067	*r* = 0.049	*r* = −0.371 *

*He*: expected heterozygosity (Table S2); Population partition: the percentage of pop 2 from the structure analysis; cluster: nominal 1 and 2 as pop 1 and 2. Asterisk (*, **, and ***) indicates significant value (*p* < 0.05, *p* < 0.01, and *p* < 0.001). The same applies in Table 3.

**Table 3:** The genetics (G) x environment (E) effect on (and) endophyte symbiosis and (on) swainsonine content.

Factors		Endophyte Infection Rate	Swainsonine Content
G	Genetic distance	*r* = 0.157 *	*r* = 0.218 ***
	Population partition	*r* = 0.014	*r* = 0.492 **
	Cluster	*r* = 0.136	*r* = 0.570 **
	He	*r* = 0.020	*r* = 0.377 *
E	Geographic distance	*r* = 0.093	*r* = 0.340 ***
	Elevation	*r* = 0.001	*r* = −0.197
	Annual precipitation	*r* = −0.261	*r* = −0.444 *
	Annul temperature	*r* = 0.008	*r* = −0.096

## Supplementary Materials

Supplementary materials can be found at https://www.mdpi.com/1422-0067/20/21/5285/s1.
