# Supplementary material for: Host Genotype and Precipitation Influence of Fungal Endophyte Symbiosis and Mycotoxin Abundance in a Locoweed
Source: Int J Mol Sci. 2019 Oct 24;20(21):5285. doi: 10.3390/ijms20215285 (PMC6862532; doi:10.3390/ijms20215285)
Supplement: Supplementary file 1 [file ijms-20-05285-s001.zip › Supplementary Figures and Tables.pdf]

Supplementary Figures and Tables

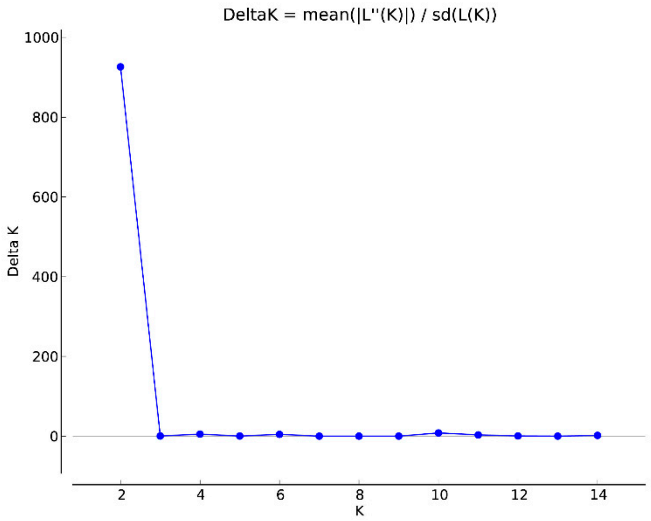

Figure A1. Determination of optimum K value for population partitioning according to Evanno *et al.* (2005). Changes of posterior probability under different assumed K value were plotted. The first significant decrease in △K indicates the best explanatory K=2, suggesting two genetically distinct populations.

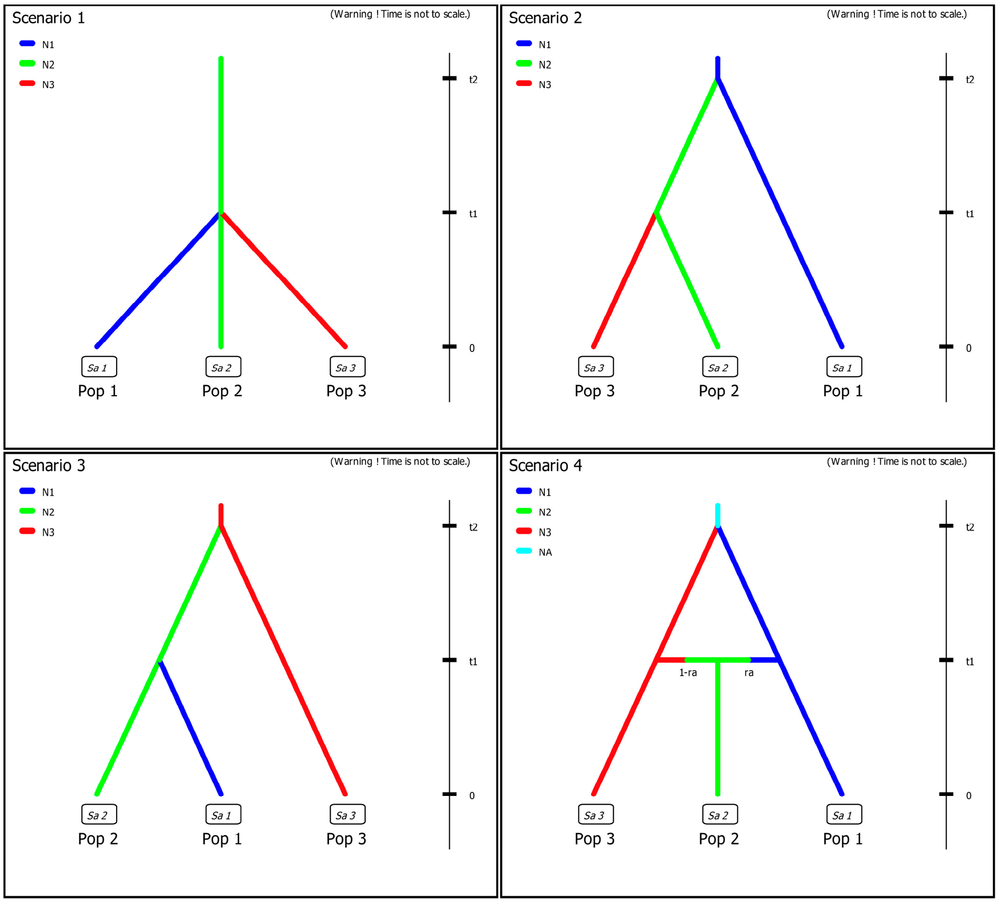

Figure A2. DIYABC analysis of evolutionary route of *O. ocherocephala*. Here, based on Figure 1A in the main text, Pop 1 and Pop 3 represent the two populations in red (northern population) and green (southern population), and Pop 2 represent GS 6 and SC6 (mixture of green and red intermediate). Posterior probability of the four scenarios were 0.0057, 0.9557, 0.0008 and 0.0377, respectively.

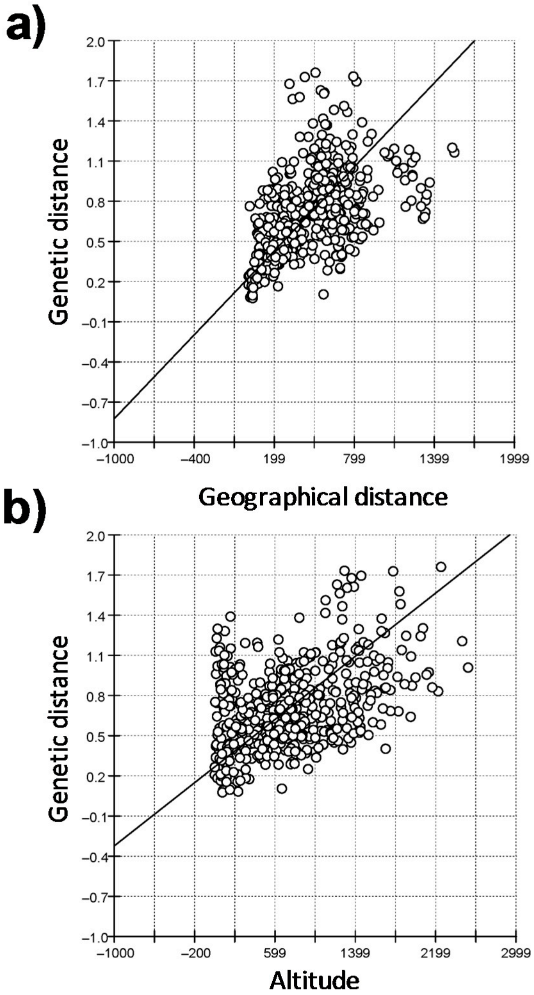

Figure A3. Scatter plots of genetic distance vs. geographical distance (panel a) and attitude distance (panel b) for pairwise population comparisons by IBD.

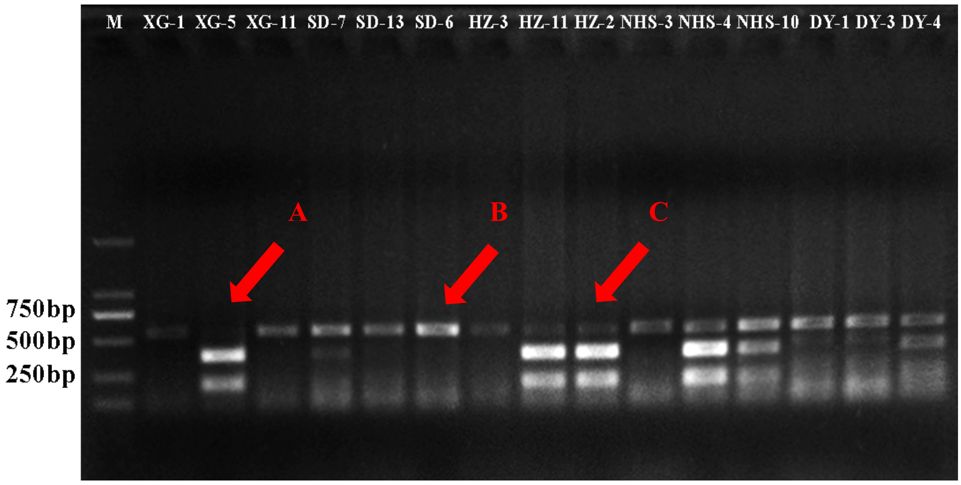

Figure A4. Agarose gel electrophoresis of PCR amplicons digested by AVA II endonuclease (partial result). Restriction digestion of PCR amplicons by AVA II. Arrows A, B, and C showed complete digestion, uncut and partial digestion, respectively.

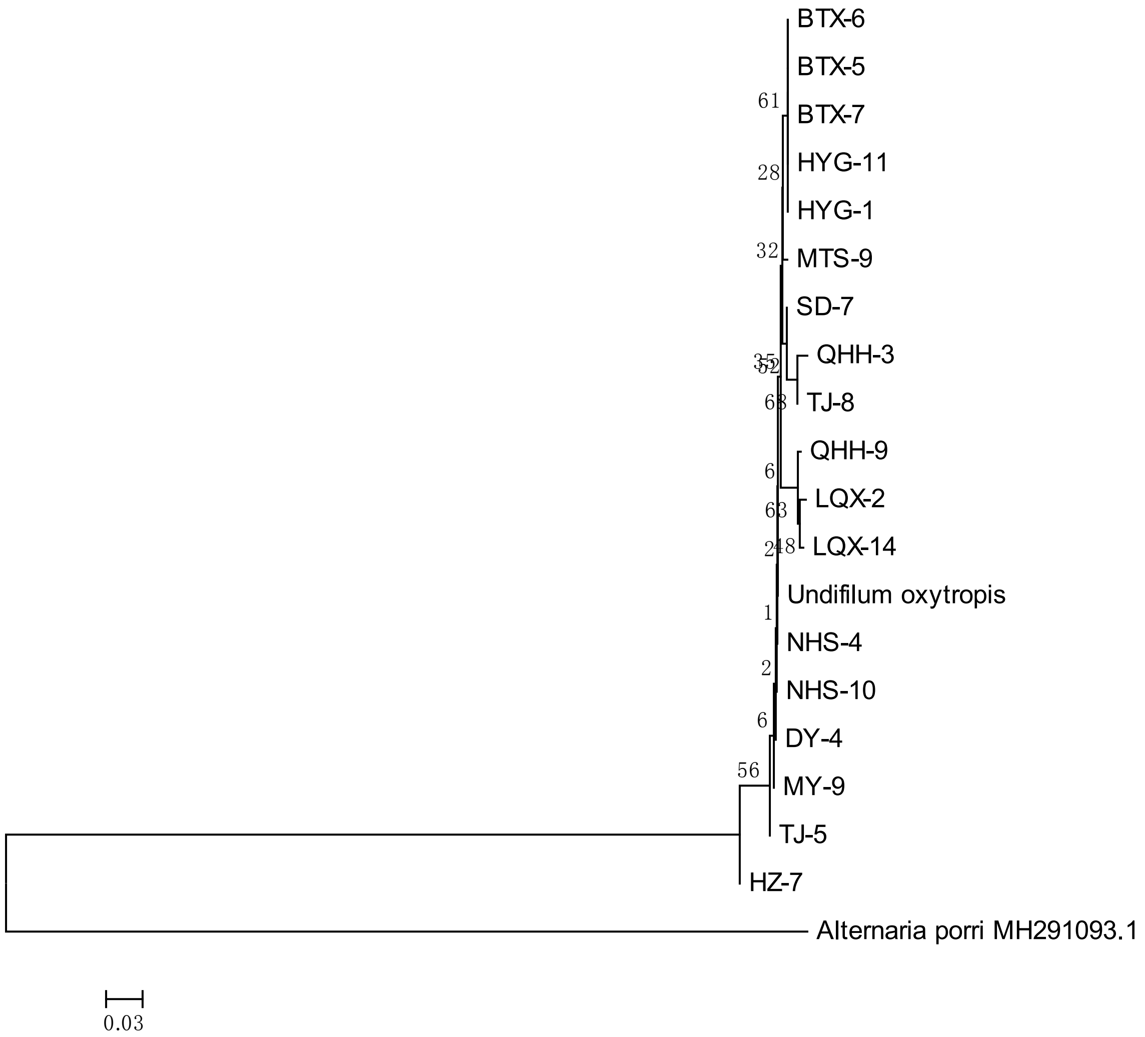

Figure A5. Tree 1. Neighbour-joining phylogenetic tree of the digested amplicons (from the partially digested samples) proved to be the *A. oxytropis* ITS sequence. Refer to Table A4 for sample names.

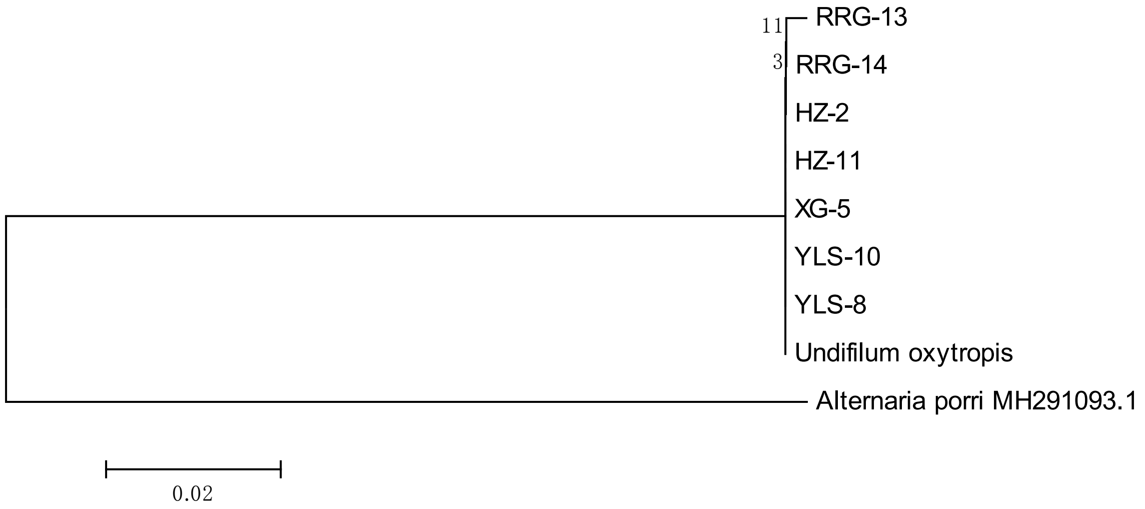

Figure A6. Tree 2. Neighbour-joining phylogenetic tree of the seven sequences which were identified to be the *A. oxytropis* ITS sequence. Refer to Table A4 for sample names.

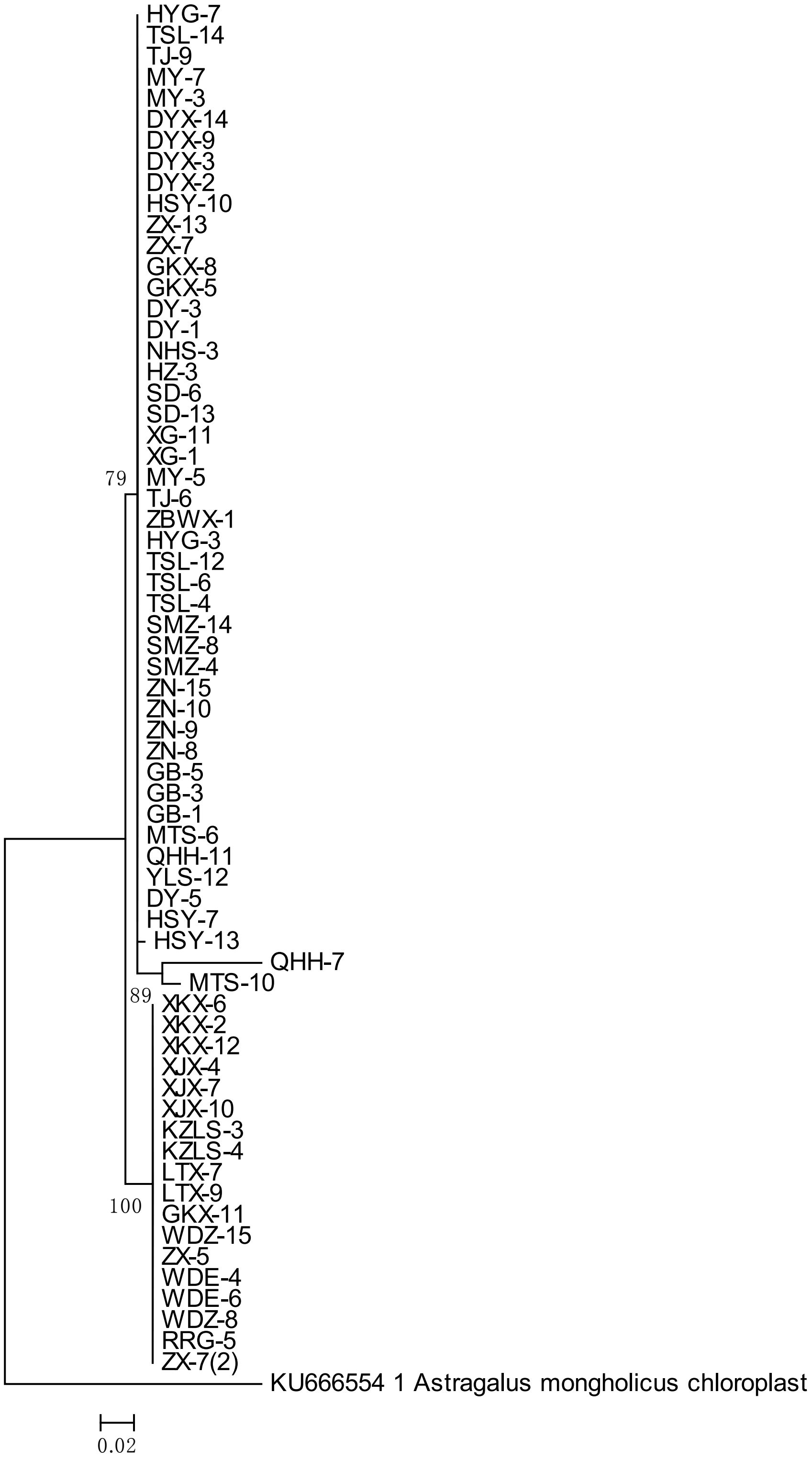

Figure A7. Tree 3. Neighbor-joining phylogenetic tree of the 65 uncut sequences using homologous *Astragalus mongholicus* chloroplast sequence as an outgroup (KU666554_1). Refer to Table A4 for sample names.

Table A1. Fourteen EST-SSR primersets and polymorphism revealed.

Locus	Repeat motif	Primer (5'-3')	Size range (bp)	*Na*	*Ne*	*Ho*	*He*	*PIC*
comp88130	(AAG)6	F:TTGGAAGGGGATTTGGGGAC<br>R:TTGCATCAGAAAATCGCCGA	256-332	25	5.71	0.410	0.826	0.80
comp78837	(AGG)6	F:TCCTAGTGTGAAACCGGTGT<br>R:TCCTCTCACATGCAACAGCA	108-126	5	1.93	0.652	0.484	0.41
comp58450	(AAT)6	F:GGTGCATCTTCTTCTTTGGCT<br>R:GACAGATCGAACGTCCCTCC	160-175	6	3.34	0.541	0.701	0.65
comp84890	(TCA)6	F:CAAGGGACTGGTCTGTCTGT<br>R: AATGTCCATGTCCGTGTGCT	230-280	19	8.73	0.302	0.887	0.87
comp87015	(GGA)8	F:CAACAAAGGTGGGGGCAATG<br>R:GGCTGGACATGTGAGCTTCT	187-220	10	3.09	0.505	0.677	0.64
comp83179	(CTCTG)7	F:TTCGCTACAAACACGAACGC<br>R:ATCGTCGCGGATTTCATCGA	137-186	19	8.49	0.359	0.884	0.87
comp88998	(CATGGT)8	F:TCCTTTGCAAGAAATAAACGCCA<br>R: GCCATTTGAGGAGCCTTTGC	174-217	8	4.44	0.422	0.776	0.74
comp90831	(GGTGTC)10	F:GGAGCTTCATCAGACTCGGT<br>R:ATCGTAAGCAGCATCCTGGG	190-236	8	3.61	0.454	0.724	0.68
comp75317	(AC)9	F:CGCCACGTATCCCAACGATA<br>R:TTCGATTTCGGCCATGACCA	239-273	18	5.29	0.441	0.812	0.79
comp68290	(AT)9	F:CCTTGGTACATGCTTGGCCT<br>R:TGTCTTGCTGACCGGATTGA	262-308	19	8.36	0.595	0.882	0.87
comp71815	(ATC)7	F:TGTTTGGGGCAATCCTCCAT<br>R:TGGAGTTAGGCATGGGTTGG	271-313	15	7.30	0.357	0.864	0.85
comp79558	(CAT)7	F:CAGCAGCCGAGTTCCTATCA<br>R: GTGCTATTCTCATTCAGAGCCA	230-257	11	2.13	0.582	0.531	0.50

comp89638	(TGA)7	F:GCCAAGCACCAAGATTGCAA<br>R:TCTCCTCCAATGGATCCCAGA	261-294	12	4.59	0.395	0.783	0.75
comp90490	(TGGAA)5	F:GATGACCGACATAGCCAGCA<br>R:TCAACAAACATCTAGAACCAGGGA	245-287	17	6.24	0.484	0.841	0.82
Mean				13.7	5.23	0.464	0.762	0.73
F statistics and Gene flow				F_{IS}: 0.176	F_{ST}: 0.253	F_{IT}: 0.385	Gene flow: 0.738	

Na: Observed number of allele; *Ne*: Effective number of alleles; *Ho*: observed heterozygosity; *He*: expected heterozygosity; *PIC:* Polymorphic information content. Locus corresponds to unigene code generated in a previous study (He, Zhuang et al. 2015).

Table A2. Sampling sites of 33 *O. ochrocephala* accessions in five provinces in China and their genetic diversity.

Accession number	Source	Accession Code	Longitude (E)	Latitude (N)	Elevation (m)	N	*Ho*	*He*
1	Nanhua Mt., Haiyuan, Ninhowgxia	NX1	105°36′49.60"	36°29′49.84"	2171	10	0.514	0.506
2	Moon Mt., Xiji, Ningxia	NX2	105°36′49.05"	36°10′29.90"	2338	15	0.519	0.560
3	Wushao Mt., Tianzhu, Gansu	GS1	102°51′27.06"	37°12′58.32"	2896	4	0.381	0.703
4	Dingyuan, Gansu	GS2	104°0′12.49"	35°57′38.28"	1780	5	0.586	0.268
5	Dayou town, Yongdeng, Gansu	GS3	102°55′08.42"	36°49′27.60"	2862	15	0.503	0.558
6	Heisongyi town, Gulang, Gansu	GS4	102°53′54.54"	37°18′02.25"	2552	15	0.471	0.614
7	Sunan, Gansu	GS5	99°41′38.57"	38°48′43.30"	2352	15	0.433	0.593
8	Luqu, Gansu	GS6	102°33′36.24"	34°09′43.40"	3529	15	0.514	0.598
9	Sunan, Gansu	GS7	100°26′06.40"	38°28′42.11"	2647	15	0.419	0.584
10	Shimen town, Tianzhu, Gansu	GS8	103°01′17.67"	36°58′44.73"	2628	15	0.534	0.540
11	Tanshanling town, Tianzhu, Gansu	GS9	102°40′26.25"	36°57′49.02"	2779	15	0.386	0.570
12	Zhuoni, Gansu	GS10	103°53′40.18"	34°50′34.12"	2194	2	0.643	0.238
13	Wanmao, Zhuon, Gansu	GS11	103°12′28.69"	34°47′20.42"	2987	15	0.450	0.671
14	Zhuaxi, Gansu	GS12	102°48′28.99"	37°11′12.39"	2861	15	0.386	0.647
15	Huangzhong, Qinghai	QH1	101°29′13.50"	36°47′13.62"	2714	2	0.321	0.607
16	Qilian Mt., Qiliang ,Qinghai	QH2	100°18′51.12"	37°57′13.62"	3780	5	0.400	0.675
17	Gonghe, Qinghai	QH3	100°42′27.60"	36°30′48.48"	3296	5	0.329	0.664
18	Lianhua Mt., Huangzhong, Qinghai	QH4	101°33′17.07"	36°29′34.99"	2747	16	0.460	0.601
19	Menyuan, Qinghai	QH5	101°15′54.41"	37°43′52.22"	2850	10	0.250	0.724
20	Gonghe, Qinghai	QH6	100°46′19.71"	36°46′13.59"	3355	15	0.376	0.688
21	Songduo, Huzhu, Qinghai	QH7	102°08′11.78"	36°43′13.42"	2599	15	0.398	0.671
22	Kuaierma, Tianjun, Qinghai	QH8	99°00'2.49"	37°25′24.16"	3610	10	0.414	0.646
23	Xigou, Minhe, Qinghai	QH9	102°44′32.67"	36°11′37.66"	2078	15	0.401	0.670
24	Geka, Daofu, Sichuan	SC1	101°16′40.77"	30°50′35.31"	3508	15	0.477	0.631

25	Kazila Mt., Yajiang, Sichuan	SC2	100°50′40.22"	29°59′52.52"	4303	10	0.457	0.549
26	Longri, Hongyuan, Sichuan	SC3	102°21′56.63"	32°27′46.86"	3557	5	0.471	0.606
27	Litang, Sichuan	SC4	100°19′16.94"	30°01′00.58"	4244	8	0.616	0.437
28	Rangkou, Hongyuan, Sichuan	SC5	102°32′13.37"	32°07′06.63"	3440	15	0.484	0.586
29	Ruoergai, Sichuan	SC6	102°39′08.40"	33°27′29.34"	3502	15	0.500	0.633
30	Tuoba, Ganzi, Sichuan	SC7	100°08′31.65"	31°35′22.35"	3630	5	0.586	0.491
31	Wengda, Seda, Sichuan	SC8	100°44′03.12"	31°51′39.16"	3442	15	0.584	0.528
32	Mengbi Mt., Xiaojin, Sichuan	SC9	102°19′16.50"	31°42′15.28"	4033	11	0.564	0.492
33	Gongbujiangda, Tibet	XZ1	93°14′03.89″	30°01′18.39″	3600	5	0.657	0.232
Mean						11	0.469	0.569

"Source" represented detailed sampling location (if possible), county name, and province name in sequential order. *Na*=Observed number of allele; *Ne*=Effective number of alleles; *Ho*: observed heterozygosity; *He*: expected heterozygosity.

Table A3. Swainsonine concentration and endophyte amount in the 33 accessions.

Accession Code	SW (‰ as DW)	Endophyte infection rate (%)
NX1	0	70.0%
NX2	0.0283±0.0015	73.3%
GS1	0	0.0%
GS2	0	0.0%
GS3	0.0062±0.0026	38.5%
GS4	0.0152±0.0025	7.0%
GS5	0.0075±0.0032	23.1%
GS6	0.0067±0.0027	40.0%
GS7	0.0062±0.0013	26.7%
GS8	0.0357±0.0042	24.9%
GS9	0.0078±0.0003	35.7%
GS10	0	0.0%
GS11	0.0350±0.0014	28.6%
GS12	0.0383±0.0023	8.3%
QH1	0.0249±0.0091	50.0%
QH2	0.0383±0.0027	60.0%
QH3	0.0050±0.0001	50.0%
QH4	0.0338±0.0022	68.8%
QH5	0.0162±0.0006	10.0%
QH6	0.0316±0.0053	86.7%
QH7	0.0362±0.0027	30.7%
QH8	0.0217±0.0052	70.0%
QH9	0.0092±0.0004	33.3%
XZ1	0	0.0%
SC1	0	0.0%
SC2	0	0.0%
SC3	0	75.0%
SC4	0	0.0%
SC5	0	0.0%
SC6	0	50.0%
SC7	0	100.0%
SC8	0.0063±0.0049	0.0%
SC9	0.0136±0.0022	40.0%

Data represented mean±SEM in swainsonine concentration.

Table A4. Sequencing result of PCR amplicons from 90 randomly selected *O. ocherocephla* samples. "ITS" refers to *A. oxytropis* (isolated by culture method from the host plant in our lab) ITS sequence (573 bp, homologous to AY228650.1 isolated from locoweeds), whilst the "Chloroplast" refers to a chloroplast sequence (560 bp, KU666554.1 homologous to the *Astragalus mongholicus* chloroplast gene). Tree 1, 2 and 3 refer to the phylogenetic tree in Figure A5, A6 and A7, respectively.

Sample name	Sequencing primer	Sequencing Result	*In silico* AVA II site prediction based on sequencing result	Restriction digestion result（3h）	Tree figure No.
QHH-3	ITS5	Doboule peaks	N/A	Partial	Figure A5
QHH-3	OR1a	Doboule peaks	N/A	Partial	Figure A5
QHH-9	ITS5	Doboule peaks	N/A	Partial	Figure A5
QHH-9	OR1a	Doboule peaks	N/A	Partial	Figure A5
MTS-9	ITS5	Doboule peaks	N/A	Partial	Figure A5
MTS-9	OR1a	Doboule peaks	N/A	Partial	Figure A5
LQX-2	ITS5	Doboule peaks	N/A	Partial	Figure A5
LQX-2	OR1a	Doboule peaks	N/A	Partial	Figure A5
LQX-14	ITS5	Doboule peaks	N/A	Partial	Figure A5
LQX-14	OR1a	Doboule peaks	N/A	Partial	Figure A5
HYG-1	ITS5	Doboule peaks	N/A	Partial	Figure A5
HYG-1	OR1a	Doboule peaks	N/A	Partial	Figure A5
HYG-11	ITS5	Doboule peaks	N/A	Partial	Figure A5
HYG-11	OR1a	Doboule peaks	N/A	Partial	Figure A5
BTX-7	ITS5	Doboule peaks	N/A	Partial	Figure A5
BTX-7	OR1a	Doboule peaks	N/A	Partial	Figure A5
BTX-6	ITS5	Doboule peaks	N/A	Partial	Figure A5
BTX-6	OR1a	Doboule peaks	N/A	Partial	Figure A5
BTX-5	ITS5	Doboule peaks	N/A	Partial	Figure A5
BTX-5	OR1a	Doboule peaks	N/A	Partial	Figure A5
SD-7	ITS5	Doboule peaks	N/A	Partial	Figure A5
SD-7	OR1a	Doboule peaks	N/A	Partial	Figure A5
NHS-4	ITS5	Doboule peaks	N/A	Partial	Figure A5
NHS-4	OR1a	Doboule peaks	N/A	Partial	Figure A5
NHS-10	ITS5	Doboule peaks	N/A	Partial	Figure A5
NHS-10	OR1a	Doboule peaks	N/A	Partial	Figure A5
DY-4	ITS5	Doboule peaks	N/A	Partial	Figure A5
DY-4	OR1a	Doboule peaks	N/A	Partial	Figure A5
MY-9	ITS5	Doboule peaks	N/A	Partial	Figure A5
MY-9	OR1a	Doboule peaks	N/A	Partial	Figure A5
TJ-5	ITS5	Doboule peaks	N/A	Partial	Figure A5
TJ-5	OR1a	Doboule peaks	N/A	Partial	Figure A5
TJ-8	ITS5	Doboule peaks	N/A	Partial	Figure A5
TJ-8	OR1a	Doboule peaks	N/A	Partial	Figure A5

HZ-7	ITS5	Doboule peaks	N/A	Partial	Figure A5
HZ-7	OR1a	Doboule peaks	N/A	Partial	Figure A5
YLS-8	ITS5	ITS	Y	Partial	Figure A6
YLS-8	OR1a	ITS	Y	Partial	Figure A6
YLS-10	ITS5	ITS	Y	Partial	Figure A6
YLS-10	OR1a	ITS	Y	Partial	Figure A6
XG-5	ITS5	ITS	Y	Complete	Figure A6
XG-5	OR1a	ITS	Y	Complete	Figure A6
HZ-11	ITS5	ITS	Y	Complete	Figure A6
HZ-11	OR1a	ITS	Y	Complete	Figure A6
HZ-2	ITS5	ITS	Y	Complete	Figure A6
HZ-2	OR1a	ITS	Y	Complete	Figure A6
RRG-13	ITS5	ITS	Y	Complete	Figure A6
RRG-13	OR1a	ITS	Y	Complete	Figure A6
RRG-14	ITS5	ITS	Y	Complete	Figure A6
RRG-14	OR1a	ITS	Y	Complete	Figure A6
DY-5	ITS5	Chloroplast	N	Uncut	Figure A7
DY-5	OR1a	Chloroplast	N	Uncut	Figure A7
YLS-12	ITS5	Chloroplast	N	Uncut	Figure A7
YLS-12	OR1a	Chloroplast	N	Uncut	Figure A7
QHH-11	ITS5	Chloroplast	N	Uncut	Figure A7
QHH-11	OR1a	Chloroplast	N	Uncut	Figure A7
MTS-6	ITS5	Chloroplast	N	Uncut	Figure A7
MTS-6	OR1a	Chloroplast	N	Uncut	Figure A7
GB-1	ITS5	Chloroplast	N	Uncut	Figure A7
GB-1	OR1a	Chloroplast	N	Uncut	Figure A7
GB-3	ITS5	Chloroplast	N	Uncut	Figure A7
GB-3	OR1a	Chloroplast	N	Uncut	Figure A7
GB-5	ITS5	Chloroplast	N	Uncut	Figure A7
GB-5	OR1a	Chloroplast	N	Uncut	Figure A7
XKX-6	ITS5	Chloroplast	N	Uncut	Figure A7
XKX-6	OR1a	Chloroplast	N	Uncut	Figure A7
XKX-2	ITS5	Chloroplast	N	Uncut	Figure A7
XKX-2	OR1a	Chloroplast	N	Uncut	Figure A7
XKX-12	ITS5	Chloroplast	N	Uncut	Figure A7
XKX-12	OR1a	Chloroplast	N	Uncut	Figure A7
ZN-8	ITS5	Chloroplast	N	Uncut	Figure A7
ZN-8	OR1a	Chloroplast	N	Uncut	Figure A7
ZN-9	ITS5	Chloroplast	N	Uncut	Figure A7
ZN-9	OR1a	Chloroplast	N	Uncut	Figure A7
ZN-10	ITS5	Chloroplast	N	Uncut	Figure A7
ZN-10	OR1a	Chloroplast	N	Uncut	Figure A7
ZN-15	ITS5	Chloroplast	N	Uncut	Figure A7

ZN-15	OR1a	Chloroplast	N	Uncut	Figure A7
SMZ-4	ITS5	Chloroplast	N	Uncut	Figure A7
SMZ-4	OR1a	Chloroplast	N	Uncut	Figure A7
SMZ-8	ITS5	Chloroplast	N	Uncut	Figure A7
SMZ-8	OR1a	Chloroplast	N	Uncut	Figure A7
SMZ-14	ITS5	Chloroplast	N	Uncut	Figure A7
SMZ-14	OR1a	Chloroplast	N	Uncut	Figure A7
XJX-4	ITS5	Chloroplast	N	Uncut	Figure A7
XJX-4	OR1a	Chloroplast	N	Uncut	Figure A7
XJX-7	ITS5	Chloroplast	N	Uncut	Figure A7
XJX-7	OR1a	Chloroplast	N	Uncut	Figure A7
XJX-10	ITS5	Chloroplast	N	Uncut	Figure A7
XJX-10	OR1a	Chloroplast	N	Uncut	Figure A7
KZLS-3	ITS5	Chloroplast	N	Uncut	Figure A7
KZLS-3	OR1a	Chloroplast	N	Uncut	Figure A7
KZLS-4	ITS5	Chloroplast	N	Uncut	Figure A7
KZLS-4	OR1a	Chloroplast	N	Uncut	Figure A7
TSL-4	ITS5	Chloroplast	N	Uncut	Figure A7
TSL-4	OR1a	Chloroplast	N	Uncut	Figure A7
TSL-6	ITS5	Chloroplast	N	Uncut	Figure A7
TSL-6	OR1a	Chloroplast	N	Uncut	Figure A7
TSL-12	ITS5	Chloroplast	N	Uncut	Figure A7
TSL-12	OR1a	Chloroplast	N	Uncut	Figure A7
HYG-3	ITS5	Chloroplast	N	Uncut	Figure A7
HYG-3	OR1a	Chloroplast	N	Uncut	Figure A7
LTX-7	ITS5	Chloroplast	N	Uncut	Figure A7
LTX-7	OR1a	Chloroplast	N	Uncut	Figure A7
LTX-9	ITS5	Chloroplast	N	Uncut	Figure A7
LTX-9	OR1a	Chloroplast	N	Uncut	Figure A7
ZBWX-1	ITS5	Chloroplast	N	Uncut	Figure A7
ZBWX-1	OR1a	Chloroplast	N	Uncut	Figure A7
GKX-11	ITS5	Chloroplast	N	Uncut	Figure A7
GKX-11	OR1a	Chloroplast	N	Uncut	Figure A7
WDZ-15	ITS5	Chloroplast	N	Uncut	Figure A7
WDZ-15	OR1a	Chloroplast	N	Uncut	Figure A7
TJ-6	ITS5	Chloroplast	N	Uncut	Figure A7
TJ-6	OR1a	Chloroplast	N	Uncut	Figure A7
QHH-7	ITS5	Chloroplast	N	Uncut	Figure A7
QHH-7	OR1a	Chloroplast	N	Uncut	Figure A7
MY-5	ITS5	Chloroplast	N	Uncut	Figure A7
MY-5	OR1a	Chloroplast	N	Uncut	Figure A7
MTS-10	ITS5	Chloroplast	N	Uncut	Figure A7
MTS-10	OR1a	Chloroplast	N	Uncut	Figure A7

XG-1	ITS5	Chloroplast	N	Uncut	Figure A7
XG-1	OR1a	Chloroplast	N	Uncut	Figure A7
XG-11	ITS5	Chloroplast	N	Uncut	Figure A7
XG-11	OR1a	Chloroplast	N	Uncut	Figure A7
SD-13	ITS5	Chloroplast	N	Uncut	Figure A7
SD-13	OR1a	Chloroplast	N	Uncut	Figure A7
SD-6	ITS5	Chloroplast	N	Uncut	Figure A7
SD-6	OR1a	Chloroplast	N	Uncut	Figure A7
HZ-3	ITS5	Chloroplast	N	Uncut	Figure A7
HZ-3	OR1a	Chloroplast	N	Uncut	Figure A7
NHS-3	ITS5	Chloroplast	N	Uncut	Figure A7
NHS-3	OR1a	Chloroplast	N	Uncut	Figure A7
DY-1	ITS5	Chloroplast	N	Uncut	Figure A7
DY-1	OR1a	Chloroplast	N	Uncut	Figure A7
DY-3	ITS5	Chloroplast	N	Uncut	Figure A7
DY-3	OR1a	Chloroplast	N	Uncut	Figure A7
GKX-5	ITS5	Chloroplast	N	Uncut	Figure A7
GKX-5	OR1a	Chloroplast	N	Uncut	Figure A7
GKX-8	ITS5	Chloroplast	N	Uncut	Figure A7
GKX-8	OR1a	Chloroplast	N	Uncut	Figure A7
ZX-5	ITS5	Chloroplast	N	Uncut	Figure A7
ZX-5	OR1a	Chloroplast	N	Uncut	Figure A7
ZX-7	ITS5	Chloroplast	N	Uncut	Figure A7
ZX-7	OR1a	Chloroplast	N	Uncut	Figure A7
ZX-13	ITS5	Chloroplast	N	Uncut	Figure A7
ZX-13	OR1a	Chloroplast	N	Uncut	Figure A7
WDE-4	ITS5	Chloroplast	N	Uncut	Figure A7
WDE-4	OR1a	Chloroplast	N	Uncut	Figure A7
WDE-6	ITS5	Chloroplast	N	Uncut	Figure A7
WDE-6	OR1a	Chloroplast	N	Uncut	Figure A7
HSY-10	ITS5	Chloroplast	N	Uncut	Figure A7
HSY-10	OR1a	Chloroplast	N	Uncut	Figure A7
HSY-13	ITS5	Chloroplast	N	Uncut	Figure A7
HSY-13	OR1a	Chloroplast	N	Uncut	Figure A7
WDZ-8	ITS5	Chloroplast	N	Uncut	Figure A7
WDZ-8	OR1a	Chloroplast	N	Uncut	Figure A7
DYX-2	ITS5	Chloroplast	N	Uncut	Figure A7
DYX-2	OR1a	Chloroplast	N	Uncut	Figure A7
DYX-3	ITS5	Chloroplast	N	Uncut	Figure A7
DYX-3	OR1a	Chloroplast	N	Uncut	Figure A7
DYX-9	ITS5	Chloroplast	N	Uncut	Figure A7
DYX-9	OR1a	Chloroplast	N	Uncut	Figure A7
DYX-14	ITS5	Chloroplast	N	Uncut	Figure A7

DYX-14	OR1a	Chloroplast	N	Uncut	Figure A7
MY-3	ITS5	Chloroplast	N	Uncut	Figure A7
MY-3	OR1a	Chloroplast	N	Uncut	Figure A7
MY-7	ITS5	Chloroplast	N	Uncut	Figure A7
MY-7	OR1a	Chloroplast	N	Uncut	Figure A7
RRG-5	ITS5	Chloroplast	N	Uncut	Figure A7
RRG-5	OR1a	Chloroplast	N	Uncut	Figure A7
TJ-9	ITS5	Chloroplast	N	Uncut	Figure A7
TJ-9	OR1a	Chloroplast	N	Uncut	Figure A7
ZX-7	ITS5	Chloroplast	N	Uncut	Figure A7
ZX-7	OR1a	Chloroplast	N	Uncut	Figure A7
HYG-7	ITS5	Chloroplast	N	Uncut	Figure A7
HYG-7	OR1a	Chloroplast	N	Uncut	Figure A7
TSL-14	ITS5	Chloroplast	N	Uncut	Figure A7
TSL-14	OR1a	Chloroplast	N	Uncut	Figure A7
HSY7	ITS5	Chloroplast	N	Uncut	Figure A7
HSY7	OR1a	Chloroplast	N	Uncut	Figure A7

"ITS" refers to *U. oxytropis* (isolated by culture method from the host plant in our lab) ITS sequence (573 bp, homologous to AY228650.1 isolated from locoweeds), whilst the "Chloroplast" refers to a chloroplast sequence (560 bp, KU666554.1 homologous to the *Astragalus mongholicus* chloroplast gene). Tree 1, 2 and 3 refer to the phylogenetic tree in Figure A5, A6 and A7, respectively.
